# Effects of High-Mobility Group A Protein Application on Canine Adipose-Derived Mesenchymal Stem Cells *In Vitro*

**DOI:** 10.1155/2012/752083

**Published:** 2012-02-08

**Authors:** A. A. Ismail, S. Wagner, H. Murua Escobar, S. Willenbrock, K. A. Sterenczak, M. T. Samy, A. M. Abd El-Aal, I. Nolte, P. Wefstaedt

**Affiliations:** ^1^Small Animal Hospital, University of Veterinary Medicine Foundation, 30559 Hannover, Germany; ^2^Department of Surgery, Anesthesiology and Radiology, Faculty of Veterinary Medicine, Zagazig University, El-Sharkia, Egypt; ^3^Centre for Human Genetics, University of Bremen, 28359 Bremen, Germany

## Abstract

Multipotency and self-renewal are considered as most important features of stem cells to persist throughout life in tissues. In this context, the role of HMGA proteins to influence proliferation of adipose-derived mesenchymal stem cell (ASCs) while maintaining their multipotent and self-renewal capacities has not yet been investigated. Therefore, extracellular HMGA1 and HMGA2 application alone (10–200 ng/mL) and in combination with each other (100, 200 ng/mL each) was investigated with regard to proliferative effects on canine ASCs (cASCs) after 48 hours of cultivation. Furthermore, mRNA expression of multipotency marker genes in unstimulated and HMGA2-stimulated cASCs (50, 100 ng/mL) was analyzed by RT-qPCR. HMGA1 significantly reduced cASCs proliferation in concentrations of 10–200 ng/mL culture medium. A combination of HMGA1 and HMGA2 protein (100 and 200 ng/mL each) caused the same effects, whereas no significant effect on cASCs proliferation was shown after HMGA2 protein application alone. RT-qPCR results showed that expression levels of marker genes including KLF4, SOX2, OCT4, HMGA2, and cMYC mRNAs were on the same level in both HMGA2-protein-stimulated and -unstimulated cASCs. Extracellular HMGA protein application might be valuable to control proliferation of cASCs in context with their employment in regenerative approaches without affecting their self-renewal and multipotency abilities.

## 1. Introduction

Mesenchymal stem cells (MSCs) are considered as one source of progenitor cells for therapeutic approaches in regenerative medicine. Although MSCs are commonly derived from bone marrow (BMSCs) [1], adipose-tissue-derived MSCs (ASCs) might be used as an alternative multipotent cell source [2–4]. Similar to BMSCs, ASCs have been also evaluated for multilineage differentiation capacities including differentiation into a chondrogenic [5, 6], osteogenic [5–7], adipogenic [5–7], neurogenic [8, 9], myogenic [6, 8, 10], angiogenic [11], and cardiomyogenic [10] lineage. In contrast to bone marrow, a large amount of adipose tissue can easily be obtained via less invasive and harmful methods making the use of ASCs as a source of stem cells very attractive [12]. Furthermore, it has recently been reported that ASCs have stronger capabilities than BMSCs to maintain their phenotype and multipotency potential even after 25 passages of *in vitro* cultivation [10]. The self-renewal and multipotency characteristics through regular and organized cell division are the most important features of stem cells to persist throughout life in tissues. These features are regulated by stem-cell-specific transcription factors, so-called multipotency genes, including SOX2 [13], cMYC [14], KLF4 [15], OCT4 [13, 16], NANOG [16], UTF1 [17], and LIN28 [18]. For maintaining the self-renewal ability of MSCs, the balance of growth factors and signaling molecules is strongly required. Therefore, the recognition of the key environmental cues that regulate the phenomena of steady proliferation and self-renewal of adult stem cells is crucial for both *in vitro* and *in vivo* applications. On this occasion, recent studies have shown that HMGA proteins have the potential to maintain and influence efficiently these features [19–21]. The high-mobility group A (HMGA) proteins (formerly known as HMGI/Y) are a class of large and specialized nuclear nonhistone chromosomal architectural proteins [22, 23]. The common functional and structural motifs in this unique group are three DNA-binding domains, so-called AT hooks, which bind preferentially to short AT-rich DNA sequences and an acidic C-terminus [24]. HMGA proteins are encoded by two distinct genes, HMGA1 and HMGA2 [25]. High expression of both HMGA proteins in undifferentiated and proliferating mesenchymal cells of early embryos indicate an important role of HMGA proteins in the regulation of stem cell proliferation and differentiation [26]. HMGA1 is mainly expressed during cell differentiation, whereas HMGA2 expression is mainly present during cell growth and proliferation [27]. 

HMGA2 expression is present in human and mouse ES cells in the inner cell mass of blastocysts indicating a key role of these proteins during prenatal development and growth [26, 28]. Furthermore, it has been found that HMGA2 expression in pluripotent human ES is closely correlated to the expression of pluripotency specific genes UTF1, SOX2, and OCT4 [29]. Additionally, Eda et al. [30] have investigated that HMGA2 and LIN28 were downregulated upon upregulation of let-7, miR-9, and miR-125b microRNAs after neuronal induction of mouse P19 embryonic carcinoma cells. In contrast Nishino et al. [19] have found that self-renewal of mouse neural stem cells was maintained by downregulation of P16 (INK4a), P19 (Arf), and let-7b microRNA upon HMGA2-mediated expression. With regard to the effects of HMGA1 expression on cell proliferation, one previous *in vitro* study has demonstrated that downregulation of cMYC transcripts in gastric cancer cell lines led to an inhibition of HMGA1 expression upon the wnt3a/beta-catenin pathway finally resulting in reduced cell growth and proliferation [31]. The role of truncated HMGA1 protein has been evaluated for an increased adipocytic cell growth as well as development of human lipomas through its rearrangements [32]. Only few *in vitro* studies have been carried out to evaluate the role of HMGA proteins on proliferation of both undifferentiated embryonic stem cells as well as already differentiated cells. Caron et al. [33] have suggested that the HMGA2 transcription factor has a function in skeletal myogenesis of mouse embryonic cells. Li et al. [29] have demonstrated regulatory influences of HMGA2 protein expression on genes linked to human ES cell growth, mesenchymal stem cell differentiation, and adipogenic differentiation. Richter et al. [34] have found that HMGA1a, HMGA1b, and HMGA2 protein application *in vitro* results in a strong positive effect on proliferation of chondrocytes derived from porcine hyalin cartilage. In conclusion HMGA proteins are considered to play a crucial role in regulating the expression of many different genes important for cell growth and proliferation, especially of stem cells. On the basis of the introduced literature, one hypothesis of this study is that the proliferation rate of cultivated stem cells, for example, ASCs, can be enhanced or decreased by either HMGA2 or HMGA1 protein application without having an effect on the multipotent status of the cells. For a possible future *in vivo* usage of stem cells in context with regenerative or tumour therapies in dogs as well as in humans it is highly desirable to control their proliferation, for example, by local application of proteins at the target site. The dog represents a valuable biomedical model for evaluation of novel therapeutic approaches in human medicine and for the dog itself. Additionally, the dog genome is highly similar to the human genome, making it an ideal model organism for the development of human cell and gene therapeutic approaches [35]. Therefore, our study was conducted to evaluate the effect of HMGA Proteins on canine ASCs. However, the influence of ectopic HMGA1 and HMGA2 protein application on cultivated ASCS is so far unknown. Thus, the aim of the present study was to evaluate the *in vitro* influence of HMGA proteins on canine ASCs proliferation and self-renewal. Moreover, it intended to investigate expression levels of multipotency specific marker genes in HMGA2 stimulated and unstimulated cASCs.

## 2. Materials and Methods

### 2.1. Isolation and Cultivation of cASCs

All experiments were carried out in accordance with the German law guidelines for governing the care and use of animals [TSchG, §4(3)]. The methods used for isolation of canine adipose-derived mesenchymal stem cells (cASCs) were adapted from previously published protocols as described by Zuk et al. [6]. Briefly, adipose tissue samples were obtained from subcutaneous fat depots of healthy dogs, weighed and then minced into small pieces. In total 20 subcutaneous fat samples of 20 dogs of different breeds were harvested. These dogs underwent orthopaedic surgeries or ovariohysterectomies and had no any other evident disease. The tissue pieces were enzymatically dissociated at 37°C for 30 min with 0.026% collagenase I (Sigma, St. Louis, MO., USA). Enzyme activity was neutralized with Dulbecco's modified Eagle's medium (DMEM, Biochrom AG, Berlin) containing 10% fetal calf serum (FCS, PAA Laboratories GmbH, Austria). Afterwards, the digested fat samples were filtered using a 100 **μ**m cell strainer (BD Bioscience, Bedford, USA) to remove debris. The cell pellets obtained after centrifugation were resuspended in DMEM with 10% FCS and plated in 25 cm^2^ flasks (TPP AG, Switzerland). The cultured cells were then maintained in an incubator supplied with humidified atmosphere of 5% CO_2_ and 95% air at 37°C. Next day, the medium was exchanged in order to remove cell debris and red blood cells. Medium change was performed every second day. When the monolayer of adherent cells reached approximately 70–80% confluence; trypsinization for cell splitting was performed using trypsin-EDTA solution (0.05%/0.02%, Biochrom AG, Berlin, Germany). After centrifugation, the cell pellets were subcultured at a concentration of 3 × 10^5^ cells/mL medium on 25 cm^2^ tissue culture flasks containing DMEM/10% FCS. Cell counting was carried out by an automatic cell counter (Cellometer Nexcelom Bioscience, Lawrence, USA).

### 2.2. Multilineage Differentiation Capacity of cASCs

cASCs at passage 3 were verified for their developmental capacity to differentiate into cells of the osteogenic, adipogenic, and chondrogenic lineage. For each differentiation experiment three 25 cm^2^ tissue culture flasks were used. In addition, each differentiation experiment was repeated more than one time.

### 2.3. Osteogenic Differentiation of cASCs

For osteogenic differentiation, cASCs at passage 3 were cultured in 25 cm^2^ flasks at a concentration of 6 × 10^5^ in presence of osteogenic induction medium for 5 weeks. Osteogenic induction medium was prepared as previously described by Pittenger et al. [36]. DMEM containing 10% FCS was supplemented with dexamethasone (Dexa, # D8893, Sigma-Aldrich Chemie GmbH, Steinheim, Germany) at a concentration of 0.1 **μ**M, 2-phospho-L-ascorbic acid trisodium salts (Asc 2P; # 49752, Sigma-Aldrich Chemie) at a concentration of 50 **μ**M, and *β*-glycerophosphate disodium at a concentration of 10 mM (# 50020, Sigma-Aldrich Chemie). Osteogenic induction medium was changed every third day. Negative controls consisted of cASCs cells maintained in DMEM with 10% FCS. Before staining, the cells were washed with PBS (2 times) and fixed in 4% paraformaldehyde solution (PFA) for 10 min at room temperature, followed again by rinsing 3 times with PBS. To verify osteogenic differentiation, von Kossa staining was carried out to detect calcified extracellular matrix deposits (ECM).

### 2.4. Chondrogenic Differentiation of cASCs

For chondrogenic induction, the micromass culture technique was used [37]. cASCs at passage 2 were seeded out in 25 cm^2^ flasks (with flat cultivation surface) using chondrogenic induction medium at a concentration of 2 × 10^6^/flask. Chondrogenic induction medium consisted of DMEM, 10% FCS, 0.1 **μ**M Dexa, 50 **μ**M Asc-2p, 0.35 g/100mL D(+)-glucose (# G-7021, Sigma-Aldrich Chemie), 50 mg/mL (1x) ITS + 1 liquid media supplement (10 **μ**g/mL insulin, 5.5 **μ**g/mL transferrin, 5 ng/mL selenium, 0.5 mg/mL bovine albumin, 4.7 **μ**g/mL linoleic acid) (# I-2521, Sigma-Aldrich Chemie), and 10 ng/mL transforming growth factor (rhTGF-*β*1; #100-21, PeproTech GmbH, Hamburg, Germany). After seeding, cells were allowed to adhere for 4 hours (37°C, humidified atmosphere, 5% CO_2_). Afterwards, the cells were maintained in induction medium for 21 days. The culture medium was changed every third day. Negative controls consisted of cASCs maintained in noninductive basal medium. Fixation of the cells was carried out as described above. Chondrogenic induction was confirmed by Alcian blue staining for chondrocyte-specific proteoglycans.

### 2.5. Adipogenic Differentiation of cASCs

For adipogenic induction, cASCs were seeded in 25 cm^2^ flasks at a concentration of 2 × 10^6^ cells/flask using DMEM medium containing 10% FCS. After a cultivation period of 3 h the medium was discarded, followed by a cultivation period of 72 h in adipogenic induction medium. Adipogenic induction medium consisted of DMEM supplemented with 10% FCS, 1 **μ**M dexa, 10 **μ**g/mL insulin (# I-6634, Sigma-Aldrich Chemie), 100 **μ**M indomethacin (#I-7378, Sigma-Aldrich Chemie), and 500 **μ**M isobutylmethylxanthine (#I-5879, Sigma-Aldrich Chemie GmbH, Steinheim, Germany). After removal of adipogenic induction medium the cells were cultivated for 24 h in adipogenic maintenance medium consisting of DMEM basal medium, 10% FCS, and 10 **μ**g/mL insulin. Cells were then cultivated again in adipogenic induction medium for 72 h, followed by another 24 h in adipogenic maintenance medium. This cycle was repeated four times in total (16 d), followed by one week of cultivation in adipogenic maintenance medium. Negative controls consisted of cASCs maintained in noninductive basal medium for 23 days. Fixation of the cells was carried out as described above. Adipogenic differentiation was confirmed by intracellular lipid droplets accumulation after Oil Red O staining.

### 2.6. In Vitro cASCs Proliferation Assay

cASCs were harvested by trypsinization and resuspended in fresh tissue culture medium as described above. The viable cell percentage from the harvested cells was calculated after trypan blue staining by an automatic cell counter (Cellometer Nexcelom Bioscience, Lawrence, USA). The cell concentration used for the proliferation assay was adjusted to 7 × 10^3^ cells/100 **μ**L medium. Liquid HMGA protein solution of HMGA1 (#:TP301458, AMS Biotechnology, Ltd, Germany) and HMGA2 (#H00008091-Q01, Abnova, Germany) were added to the DMEM culture medium with 10% FCS at concentrations of 10, 50, 100, and 200 ng/mL. Furthermore a combination of HMGA1 and HMGA2 (100 and 200 ng/mL each) was used for cASC stimulation. The negative control condition consisted of unstimulated cASCs. To achieve the correct protein concentrations in the culture medium, directly before usage, stock solutions with protein concentrations of 1 **μ**g/mL DMEM culture medium (with 10% FCS) were prepared. The desired concentration of HMGA proteins was achieved by further dilution of the stock solution with DMEM (with 10% FCS). Proliferation of stimulated and unstimulated cASCs was evaluated using a bromodeoxyuridine (BrdU) cell proliferation enzyme-linked immunosorbent assay (ELISA kit; #11647229001, Roche Diagnostics GmbH, Mannheim, Germany) according to the manufacturer's instructions. For each of the analyzed protein concentrations cASCs were seeded out in eight separate wells of a 96-well cell culture microtiter plate (#353075, Microtest-Falcon, Becton Dickinson Biosciences, USA) at a concentration of 7 × 10^3^ cells per well (100 **μ**L of cell suspension). Cells were incubated for 3 hours (95% humidified air, 37°C, 5% CO_2_) for cell adhesion, followed by the addition of the corresponding amounts of each protein. After a coculturing period of 24 hours (95% humidified air, 37°C, 5% CO_2_), 10 **μ**L/well of BrdU labeling solution (final concentration; 10 **μ**m/well BrdU) was added, followed by reincubation at 37°C and 5% CO_2_ for another 24 h. During this labeling period, the pyrimidine analogue BrdU was incorporated into the DNA of the proliferating cASCs. After 48 hours as a total treatment period of HMGA protein, the culture medium was carefully removed. Afterwards, the cells were fixed and the DNA was denatured in one step by adding FixDenat solution (200 **μ**L/well). The cells were then incubated for half an hour at room temperature. The denaturation of the DNA was necessary to improve the accessibility of the incorporated BrdU for detection by the antibody. FixDenat solution was removed thoroughly via careful pipetting. Anti-BrdU-conjugate-peroxidase (Anti-BrdU-POD) working solution was then added in an amount of 100 **μ**L/well. The anti-BrdU-POD binds to the BrdU incorporated in newly synthesized, cellular DNA. After 90 minutes incubation at RT, the antibody conjugate was removed. Afterwards, the cells were rinsed three times with washing solution (PBS; 300 **μ**L/well). The immune complexes were detected by the subsequent substrate reaction. Finally the substrate solution (TMB; tetramethyl-benzidine) was added to the cells (100 **μ**L/well), and the cells were incubated at RT for another 25 minutes. The photometric absorbance values of the reaction products were measured using a scanning multiwell spectrophotometer (Synergy 2 Multi-Mode Microplate Reader, Biotek Instruments GmbH. Germany) at 370 nm and a reference wavelength of 492 nm at 10 time points between 0 and 30 minutes. The blanked 370 nm data minor 497 nm were plotted against the timescale. The maximal slope of the absorbance curve was calculated and used for statistical comparisons. The absorbance values directly correlate to the amount of synthesized DNA and the corresponding number of proliferating cells in the respective microcultures. Absorbance values for the negative control condition were normalized to 100%. Values of all other conditions were converted, respectively.

### 2.7. Stimulation of cASCs with HMGA2 Protein for Subsequent RT-qPCR

According to the above-mentioned protocols cASCs were seeded in six-well tissue culture plates at a concentration of 2 × 10^4^ cells/well in DMEM (volume/well:1000 **μ**L) supplemented with 10% FCS. Cells were allowed to adhere for 4 hours. Afterwards cASCs were stimulated in triplicates with HMGA2 supplemented medium at a concentration of 50 and 100 ng HMGA2/mL culture medium (volume/well:1000 **μ**L). Unstimulated cASCs served as control. After incubation for 48 hours (humidified atmosphere, 5% CO_2_, 37°C) the cells were harvested by trypsinization, followed by centrifugation at 800 ×g for 10 minutes. The cell pellet was used for subsequent RT-qPCR.

### 2.8. Relative Quantitative Real-Time Polymerase Chain Reaction (RT-qPCR)

#### 2.8.1. RNA Isolation and cDNA Synthesis for Transcript Characterization

Total RNA was isolated from unstimulated as well as HMGA2 stimulated cASCs (100, 200 ng) using NucleoSpin miRNA (#740971.250; Macherey & Nagel GmbH, Düren. Germany) isolation kit according to the manufacturer's instructions. The concentration and purity of the isolated RNA was verified by the OD260/280 nm absorption ratio using the Multi-Mode Microplate Reader SynergyTM2 and Gene5TM microplate software (Biotek Instruments, inc, USA). cDNA was synthesised using Quantitect Reverse Transcription Kit (#205313; Qiagen, Hilden, Germany) following the manufacturer's protocol. For the reverse transcription, 250 ng of total RNA were used for the relative characterization of mRNA transcript levels of Klf4, HMGA2, SOX2, cMYC, NANOG, and Pou5F/OCT4.

#### 2.8.2. RT-qPCR

RT-qPCR was carried out using the Master Cycler Gradient Cycler (Eppendorf, Germany). The canine Hypoxanthine Phosphoribosyl Transferase-1 (HPRT1) [38] and Glucuronidase Beta transcript (GUSB) [39] were used as endogenous reference genes. The amplification was carried out in a total reaction volume of 20 **μ**L using the TaqMan Universal PCR Master Mix (# 4304437; Applied Biosystems, Darmstadt, Germany). For establishing the canine HMGA2- and Klf4-Assays, the primers and probes were designed by the software SeqMan Pro and Editseq 7.1.0 DNASTAR, Inc. (Lasergene MADISON, USA) using the murine KLF4 mRNA NM_010637, human KLF4 mRNA NM_004235 (http://www.ncbi.nlm.nih.gov/), and canine KLF4 cDNA ENSCAFT00000004467 (http://www.ensembl.org/). The designed primers and probes related to KLF4 and HMGA2 mRNA were supplied by Biomers (Ulm, Germany). The Primer and probe sequences of all other examined transcripts were synthesized by Applied Biosystems (Darmstadt, Germany). For amplification of gene transcripts, 500 nM of each primer and 200 nM of the corresponding fluorogenic probe were used. Form each sample 2 **μ**L cDNA were used as template. All reactions were carried out on the same RT-qPCR plate. PCR cycles were carried out starting with 2 min for activation at 50°C and 10 min at 95°C for template denaturation, followed by 40 cycles with 15 s at 95°C and 1 min at 60°C. All samples were measured in triplicate and for each run nontemplate controls and nonreverse transcriptase control reactions were included. The quotient of the threshold cycles for the analysed multipotency marker genes was set in relation to the expression level of the housekeeping genes HPRT1 and GUSB using the ΔΔ cycle threshold (ΔΔCT) method. Gene expression levels of the marker genes in HMGA2 stimulated cASCs were normalised to the expression level in unstimulated cASCs.

#### 2.8.3. Statistics

The statistical analyses were performed using GraphPad Prism 4 software (GraphPad Software, La Jolla, CA). The obtained data were expressed as means ± SD. Differences between groups were analyzed using a one-way ANOVA and a Tukey test for post hoc comparisons. The significance levels were set at *P* < 0.05, *P* < 0.01 and *P* < 0.001.

## 3. Results

### 3.1. Morphology of Canine ASCs

**Figure 1:**
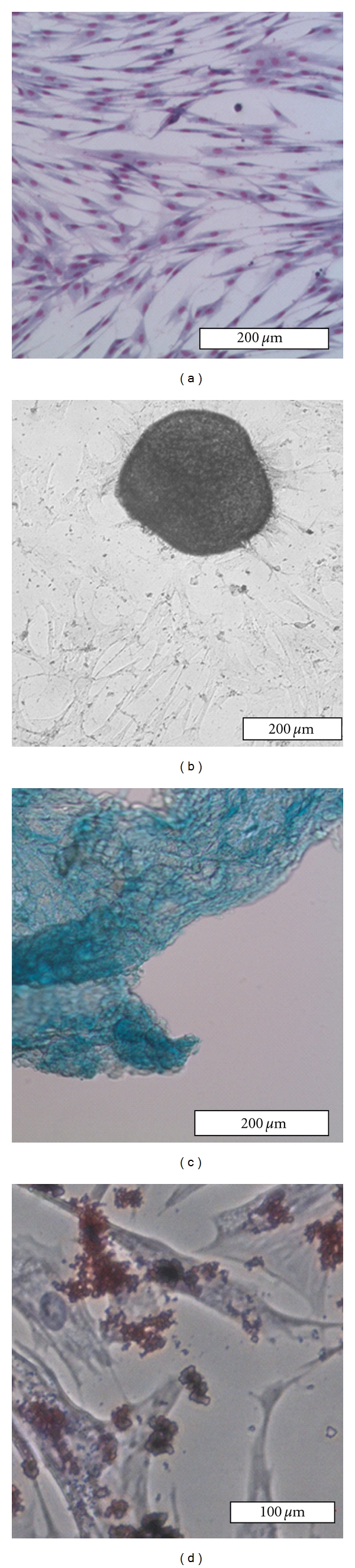
Example of Diff-Quik stained undifferentiated cASCs with flattened fibroblast-like morphology (a). After 5 weeks of osteogenic induction, von Kossa staining was positive, showing large black calcifying nodules (b). Chondrogenic differentiated cASCs show polygonal morphology after 21 days of induction in addition to positive Alcian blue staining of the chondrocyte-specific glycosaminoglycans (c). In (c) an adherent double cell layer is to be seen. Adipogenic induction could be shown 23 days after induction by positive red lipid droplets after Oil Red O staining (d). Scale bar: 200 **μ**m (a–c) and 100 **μ**m (d).

After 24 hours of cultivation, most of the seeded cells were remaining nonadherent in suspension. After washing to remove these cells, few adhered single cells or cell aggregates were observed. Initially adherent cells grew into spindle or fibroblast-like cells, which then developed into visible colonies after 3–5 days in culture. Furthermore, formation of cell-cell contacts and proliferation could be observed. After a cultivation period of two weeks adherent cells had proliferated to near confluence in 25 cm^2^ flasks. After the first passage, the fibroblast-like shape became more regular and could be maintained throughout further subcultivation (Figure 1(a)).

### 3.2. Demonstration of Multilineage Potential of cASCs

cASCs isolated from subcutaneous fat were evaluated for their ability to differentiate into cells with an osteogenic (Figure 1(b)), chondrogenic (Figure 1(c)), and adipogenic (Figure 1(d)) phenotype after cultivation in a respectively supplemented medium (see below).

### 3.3. Osteogenic Differentiation

After two weeks of cultivation in presence of osteogenic induction medium, cells changed from a fibroblastic phenotype to a more polygonal appearance. Furthermore, a small amount of the calcium deposition, normally indicative for osteogenesis, could be detected. Additionally, osteogenic differentiation was confirmed by positive von Kossa staining. Within a cultivation period of 5 weeks after induction of the cells continued to proliferate actively and formed cell aggregates with an increased amount of calcified extracellular matrix and mineralized nodules (Figure 1(b)). These findings were absent in cultures of unstimulated cASCs which maintained their fibroblast-like appearance and did not form cell aggregates (Figure 1(a)). Furthermore, no calcified ECM deposition could be observed in undifferentiated cells.

### 3.4. Chondrogenic Differentiation

Chondrogenic differentiation of cASCs was achieved after 3 weeks of micromass culture in chondrogenic induction medium. At this timepoint chondrocyte-differentiated cASCs showed a polygonal morphology. Furthermore, chondrogenic induction was confirmed by positive Alcian blue staining which is indicative for the presence of chondrocyte-specific proteoglycans (Figure 1(c)). Positive Alcian blue staining was absent in unstimulated cASCs (Figure 1(a)).

### 3.5. Adipogenic Differentiation

Adipogenic induction of cASCs was completed after 23 days of cultivation in adipogenic induction medium. Adipogenic cells showed a larger cell morphology with an accumulation of red intracellular lipid droplets after Oil Red O staining (Figure 1(d)). Positive Oil Red O staining and changed cell morphology were absent in unstimulated cASCs (Figure 1(a)).

### 3.6. Effect of HMGA Proteins on Proliferation Rate of cASCs

**Figure 2:**
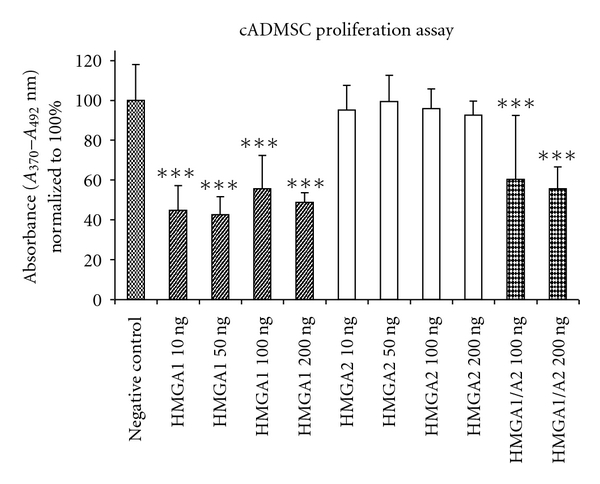
Proliferation test of cASCs stimulated with HMGA proteins. Absorbance values were normalized to 100% in the negative control condition, significance levels are given for comparisons versus negative control condition. HMGA1 at all used levels has significant suppressive effects on cell proliferation (*P* < 0.001). In contrast, cell proliferation in the HMGA2-treated groups resulted in no significant differences in comparison to the control group at all chosen concentrations. The combined protein application of 100 ng and 200 ng/mL shows a significant suppressive effect on cASCs proliferation (*P* < 0.001). For statistical comparisons a one-way analysis of variance and a Tukey posttest was carried out (significance levels: *P < 0.05; **P < 0.01; ***P < 0.001).

To evaluate whether HMGA proteins have an effect on proliferation of cASCs, cells were cocultured for 48 hours in presence of HMGA1 and HMGA2 proteins at different concentrations and in combination with each other. HMGA1 and HMGA2 related effects on cASC proliferation were compared with proliferation rates of non-HMGA-treated cASCs (Figure 2). No significant enhancement of cASC proliferation after HMGA2 protein application at concentrations of 10 (94.99 ± 12.44% (mean ± standard deviation)), 50 (99.46 ± 13.07%), 100 (95.79 ± 9.95%), and 200 (92.55 ± 6.95%) ng/mL could be detected in comparison to the proliferation rate of unstimulated cASCs (100 ± 18.04%). In contrast, HMGA1 protein application at concentrations of 10 (44.73 ± 12.37%), 50 (42.5 ± 9.05%), 100 (55.49 ± 16.83%), and 200 (48.7 ± 4.81%) ng/mL had a significant inhibitory effect on cASC proliferation (*P* < 0.001). Moreover, the combined application of HMGA1 and HMGA2 protein at respective concentrations of 100 (60.82 ± 32.04%) and 200 (55.62 ± 10.93%) ng/mL also resulted in a significant decrease of the cell proliferation rate (*P* < 0.001), when compared with untreated cells. Furthermore the proliferation rate of combined HMGA1- and HMGA2-treated cASCs was found to be on the same level as in HMGA1 stimulated cells in all used concentrations.

### 3.7. Expression Analyzes of Multipotency Marker Genes in HMGA2-Stimulated cASCs

**Figure 3:**
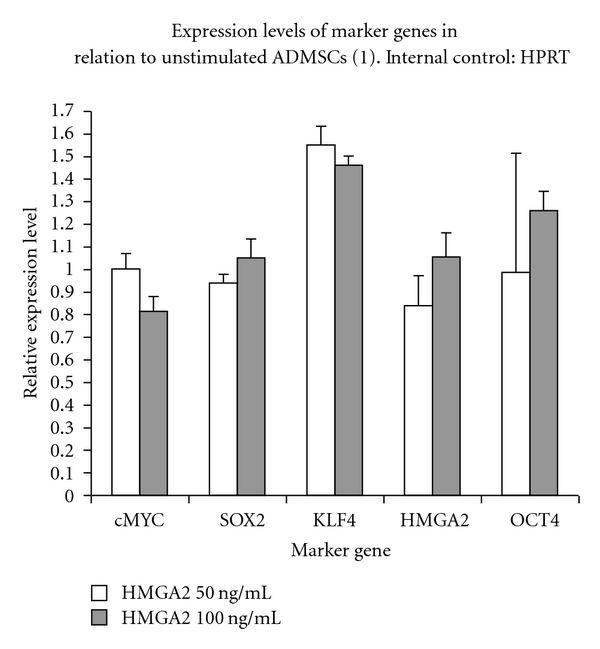
Marker gene expression in HMGA2 protein-stimulated (50, 100 ng/mL) and -unstimulated cASCs (relative expression = 1) after RT-qPCR. HPRT was used as endogenous control. All analysed genes (cMYC, SOX2, KLF4, HMGA2, and OCT4) are expressed in cASCs. No significant marker gene regulation could be detected after HMGA2 application in both concentrations.

**Figure 4:**
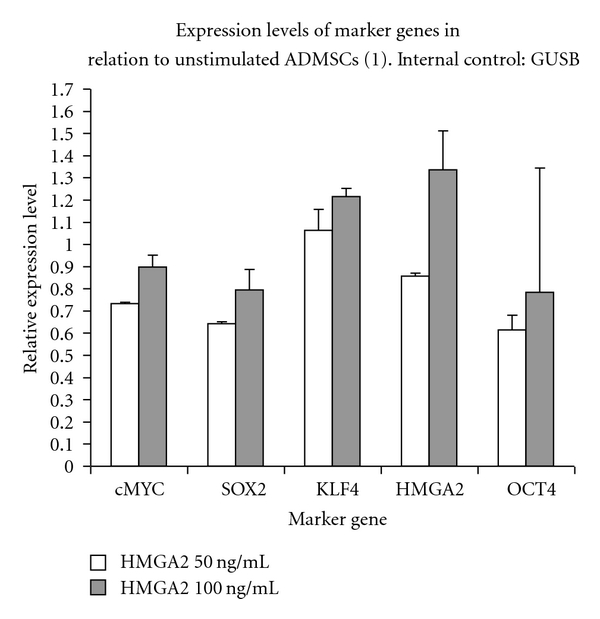
Marker gene expression in HMGA2 protein-stimulated (50, 100 ng/mL) and -unstimulated cASCs (1) after RT-qPCR. GUSB was used as endogenous control. All analysed genes (cMYC, SOX2, KLF4, HMGA2, and OCT4) are expressed in cASCs. No significant marker gene regulation could be detected after HMGA2 application in both concentrations.

Relative expression of all analyzed marker genes in unstimulated cASCs was normalized to 1 (Figures 3 and 4) and gene expression in the 50 and 100 ng/mL stimulated cASC condition was normalized accordingly. In case of the experiments in which HPRT1 (Figure 3) was used as endogenous control, KLF4 expression higher in 50 (1.55 ± 0.09) and 100 ng/mL (1.46 ± 0.04; *P* < 0.05) HMGA2-treated cASCS than in ustimulated cASCs. However, these differences were not significant. OCT4 expression in relation to HPRT1 in stimulated cASCs was measured on the same level as in unstimulated cASCs after 50 (0.99 ± 0.53; *P* > 0.05) and 100 ng/mL (1.26 ± 0.09; *P* > 0.05) HMGA2 treatment. No significant differences were found for SOX2, cMYC, and HMGA2 expression in relation to HPRT1 in cASCs after 50 (SOX2: 0.94 ± 0.04, *P* > 0.05; cMYC: 1.00 ± 0.07, *P* > 0.05; HMGA2: 0.83 ± 0.13, *P* > 0.05) and 100 ng/mL (SOX2: 1.05 ± 0.09, *P* > 0.05; cMYC: 0.81 ± 0.07, *P* > 0.05; HMGA2: 1.05 ± 0.11, *P* > 0.05) HMGA2 stimulation in comparison to unstimulated cASCs.

In case of GUSB (Figure 4) as endogenous control no significant differences with regard to KLF4 expression could be detected in cASCs after 50 (1.06 ± 0.09; *P* > 0.05) and 100 ng/mL (1.22 ± 0.04; *P* > 0.05) HMGA2 stimulation in comparison to unstimulated cells. OCT4 expression in relation to GUSB was slightly but not significantly lower in 50 (0.61 ± 0.07; *P* > 0.05) and 100 ng/mL (0.78 ± 0.56; *P* > 0.05) HMGA2-treated cASCs in comparison to unstimulated cells. No significant differences were found for SOX2, cMYC and HMGA2 expression in relation to HPRT1 in cASCs after 50 (SOX2: 0.64 ± 0.01, *P* > 0.05; cMYC: 0.73 ± 0.01, *P* > 0.05; HMGA2: 0.86 ± 0.02, *P* > 0.05) and 100 ng/mL (SOX2: 0.79 ± 0.09, *P* > 0.05; cMYC: 0.90 ± 0.05, *P* > 0.05; HMGA2: 1.33 ± 0.18, *P* > 0.05) HMGA2 stimulation in comparison to unstimulated cASCs.

## 4. Discussion

In the field of tissue engineering, a setup of well-defined and reproducible culture conditions that will allow for large-scale expansion of stem cells while providing efficient self-renewal and maintenance of multipotency is critical. The key factor in terms of multipotency and self-renewal characteristics of stem cells is the control by the expression of several unique multipotency-associated marker genes as for example, KLF4, cMYC, SOX2, HMGA2, and OCT4 [13–16, 40]. 

The present study was focused on the characterization of multipotent effects of HMGA protein application on cultivated cASCs. These adipose-tissue-derived cells have several benefits for clinical applications, providing an autologous source of adult stem cells for therapeutic approaches [41]. In general the isolation of these cells from mammalian tissues is well established as well as the possibility to expand the cells* in vitro. *Thereby, the potential of cASCs to differentiate into osteogenic, adipogenic, and chondrogenic lineages is preserved confirming the stem-cell-like character of the cells kept* in vitro*. Multilineage differentiation capacity could also be demonstrated by histologic analyses for cASCs used in this study. As the differentiation procedure is a well-established technique [36, 37] further negative controls using, for example, Alcian blue stained undifferentiated cASCs are not presented, which is, however a shortcoming of the here presented study. Recently it could be shown, that adult cASCs do not lose their stem cell features after cryopreservation [42]. Besides ASCs [5, 6], these facts are also valid for mammalian BMSCs [43]. Previous studies demonstrated that the HMGA proteins HMGA1 and HMGA2 are involved in various cellular processes such as proliferation [24] and senescence [44]. In addition, they are highly expressed in undifferentiated cells during embryogenesis and downregulated in differentiated cells [28]. Consequently, in the presented study proliferative effects as well as effects on regulation on stem cell marker genes of cASCs were characterized *in vitro*. Our data indicated that HMGA1 protein at concentrations of 10 ng to 200 ng/mL culture medium as well as a combined protein application of HMGA1 and HMGA2 (100 and 200 ng/mL) resulted in significant suppressive effects on cASCs proliferation after 48 hours of cocultivation. These findings are contrary to an earlier study which reported a reduced cell growth and proliferation due to a decreased HMGA1 expression in gastric cancer cell lines [31]. An explanation for the different response of cASCs to ectopic HMGA1 application might be that functional pathways in cancer cell lines are often not comparable to the pathways of physiological somatic cells or stem cells. In the present study no significant difference of cASC proliferation in comparison to untreated cASCs could be found after HMGA2 protein application at all used concentrations. In contrast, Richter et al. [34] were able to demonstrate that HMGA1a, HMGA1b, and HMGA2 proteins have a significant positive effect on the proliferation rate of chondrocyte monolayers *in vitro* after 24 hours of incubation. In another recently published study, Richter et al. found positive proliferative effects of HMGA2-derived fragments on proliferation of cultured porcine chondrocytes and canine ASCs [34, 45]. However, higher doses of HMG proteins ranging from 1 **μ**g to over 100 **μ**g/mL were used in the studies of Richter et al. [34, 45]. In the here presented study lower doses of HMG protein were used according to the studies performed with the HMGA sister protein HMGB1 demonstrating biologic effects at concentrations of 10–200 ng/mL protein application [46, 47]. However, in this context it has to be kept in mind that HMGB1 extracellular signaling is well characterised being mediated by TLR and RAGE receptors. In contrast to this, a detailed characterisation of extracellular HMGA receptors is still missing. To our knowledge, only few studies have been carried out to verify how HMGA proteins affect multipotency maintenance and proliferation in adult stem cells. Recently, Nishino et al. [19] have reported that HMGA2 protein knockdown led to inhibition of neural stem cell proliferation and self-renewal of fetal and young-adult mice but not old-adult mice. These findings indicate that HMGA2 might have a proliferative effect only on stem cells at early stages. This hypothesis is supported by the results of our *in vitro* study in which also no proliferative effects of HMGA2 could be found. Nevertheless it has to be stated that in our study an extracellular protein application was used, whereas in the study of Nishino et al., HMGA2 expression was knocked out [19]. In addition, microRNA let-7b has been reported to regulate self-renewal of neural stem cells through targeting on HMGA2 protein and TLX receptor [19, 48]. Our results reported here using real-time qPCR revealed that HMGA2 protein can contribute to maintain self-renewal and multipotency of cASCs as no significant effects on gene regulation of multipotency marker genes could be found. HMGA2 protein-stimulated and -unstimulated cASCs showed no significant differences in relative expression levels of the marker genes KLF4, SOX2, OCT4, cMYC, and HMGA2 in cASCs. In this context it has to be stated that the OCT4 signal was detectable only at late PCR cycles. Thus, results for this gene have to be looked at critically. However, for reasons of a complete description of the gene panel the OCT4 data is presented. A major limitation of the study is the missing data of marker gene expressions in cASCs after HMGA1 protein application. Therefore, future studies will have to investigate whether the inhibitory effect on cASC proliferation is accompanied by a changed or unaltered expression of multipotency marker genes. As another result, it could be demonstrated that the suppressive effects of HMGA1 protein application on cASC proliferation cannot be blocked by additional HMGA2 application. In case of the combined HMGA1 and HMGA2 application in a concentration of 100 ng/mL each, a high-standard deviation of the measurement values is evident. However, it would be interesting to investigate the effects of HMGA1 protein application on gene expression of the mentioned marker genes and to check for HMGA1 specific receptors on cASCs. Also, multilineage differentiation capacity of HMGA protein-stimulated ASCs remains to be investigated in further studies.

## 5. Conclusions

In conclusion, HMGA proteins remain interesting candidate proteins for influencing ASC proliferation without altering multipotency gene expression. Further studies focussing on higher concentrations of HMGA protein application on cultured ASCs are needed to fully study the effects on proliferation and marker gene expression. Characterizing the role of HMGA protein on self-renewal and multipotency of ASCs might be providing novel insights for a controlled and efficient application of these cells in regenerative approaches. Especially the possibility to control proliferation of tumour stem cells would be of great advantage in this context.

##  Authors' Contribution

A. A. Ismail and S. Wagner contributed equally to the development of the paper.

## References

[1] Leonardi E, Devescovi V, Perut F, Ciapetti G, Giunti A (2008). Isolation, characterisation and osteogenic potential of human bone marrow stromal cells derived from the medullary cavity of the femur. *La Chirurgia Degli Organi di Movimento*.

[2] Puissant B, Barreau C, Bourin P (2005). Immunomodulatory effect of human adipose tissue-derived adult stem cells: comparison with bone marrow mesenchymal stem cells. *British Journal of Haematology*.

[3] Hattori H, Sato M, Masuoka K (2004). Osteogenic potential of human adipose tissue-derived stromal cells as an alternative stem cell source. *Cells Tissues Organs*.

[4] Romanov YA, Darevskaya AN, Merzlikina NV, Buravkova LB (2005). Mesenchymal stem cells from human bone marrow and adipose tissue: isolation, characterization, and differentiation potentialities. *Bulletin of Experimental Biology and Medicine*.

[5] Neupane M, Chang CC, Kiupel M, Yuzbasiyan-Gurkan V (2008). Isolation and characterization of canine adipose-derived mesenchymal stem cells. *Tissue Engineering—Part A*.

[6] Zuk PA, Zhu M, Mizuno H (2001). Multilineage cells from human adipose tissue: implications for cell-based therapies. *Tissue Engineering*.

[7] Shi YY, Nacamuli RP, Salim A, Longaker MT (2005). The osteogenic potential of adipose-derived mesenchmal cells is maintained with aging. *Plastic and Reconstructive Surgery*.

[8] Lin Y, Chen X, Yan Z (2006). Multilineage differentiation of adipose-derived stromal cells from GFP transgenic mice. *Molecular and Cellular Biochemistry*.

[9] Safford KM, Hicok KC, Safford SD (2002). Neurogenic differentiation of murine and human adipose-derived stromal cells. *Biochemical and Biophysical Research Communications*.

[10] Zhu Y, Liu T, Song K, Fan X, Ma X, Cui Z (2008). Adipose-derived stem cell: a better stem cell than BMSC. *Cell Biochemistry and Function*.

[11] Tang YL, Zhao Q, Zhang YC (2004). Autologous mesenchymal stem cell transplantation induce VEGF and neovascularization in ischemic myocardium. *Regulatory Peptides*.

[12] Tong ML, Martina M, Hutmacher DW, Hui JHPO, Eng HL, Lim B (2007). Identification of common pathways mediating differentiation of bone marrow- and adipose tissue-derived human mesenchymal stem cells into three mesenchymal lineages. *Stem Cells*.

[13] Rizzino A (2009). Sox2 and Oct-3/4: a versatile pair of master regulators that orchestrate the self-renewal and pluripotency of embryonic stem cells. *Wiley Interdisciplinary Reviews*.

[14] Smith KN, Singh AM, Dalton S (2010). Myc represses primitive endoderm differentiation in pluripotent stem cells. *Cell Stem Cell*.

[15] Chan KKK, Zhang J, Chia NY (2009). KLF4 and PBX1 directly regulate NANOG expression in human embryonic stem cells. *Stem Cells*.

[16] Jones MB, Chu CH, Pendleton JC (2010). Proliferation and pluripotency of human embryonic stem cells maintained on type i collagen. *Stem Cells and Development*.

[17] Kooistra SM, van den Boom V, Thummer RP (2010). Undifferentiated embryonic cell transcription factor 1 regulates ESC chromatin organization and gene expression. *Stem Cells*.

[18] Richards M, Tan SP, Tan JH, Chan WK, Bongso A (2004). The transcriptome profile of human embryonic stem cells as defined by SAGE. *Stem Cells*.

[19] Nishino J, Kim I, Chada K, Morrison SJ (2008). Hmga2 promotes neural stem cell self-renewal in young but not old mice by reducing p16Ink4a and p19Arf expression. *Cell*.

[20] Monzen K, Ito Y, Naito AT (2008). A crucial role of a high mobility group protein HMGA2 in cardiogenesis. *Nature Cell Biology*.

[21] Sanna S, Jackson AU, Nagaraja R (2008). Common variants in the GDF5-UQCC region are associated with variation in human height. *Nature Genetics*.

[22] Bustin M (2001). Revised nomenclature for high mobility group (HMG) chromosomal proteins. *Trends in Biochemical Sciences*.

[23] Grosschedl R, Giese K, Pagel J (1994). HMG domain proteins: architectural elements in the assembly of nucleoprotein structures. *Trends in Genetics*.

[24] Reeves R, Edberg DD, Li Y (2001). Architectural transcription factor HMGI(Y) promotes tumor progression and mesenchymal transition of human epithelial cells. *Molecular and Cellular Biology*.

[25] Sgarra R, Rustighi A, Tessari MA (2004). Nuclear phosphoproteins HMGA and their relationship with chromatin structure and cancer. *FEBS Letters*.

[26] Tay Y, Peter S, Rigoutsos I, Barahona P, Ahmed S, Dröge P (2010). Insights into the regulation of a common variant of HMGA2 associated with human height during embryonic development. *Stem Cell Reviews and Reports*.

[27] Vartiainen E, Palvimo J, Mahonen A, Linnala-Kankkunen A, Maenpaa PH (1988). Selective decrease in low-M(r) HMG proteins HMG I and HMG Y during differentiation of mouse teratocarcinoma cells. *FEBS Letters*.

[28] Zhou X, Benson KF, Ashar HR, Chada K (1995). Mutation responsible for the mouse pygmy phenotype in the developmentally regulated factor HMGI-C. *Nature*.

[29] Li O, Li J, Dröge P (2007). DNA architectural factor and proto-oncogene HMGA2 regulates key developmental genes in pluripotent human embryonic stem cells. *FEBS Letters*.

[30] Eda A, Tamura Y, Yoshida M, Hohjoh H (2009). Systematic gene regulation involving miRNAs during neuronal differentiation of mouse P19 embryonic carcinoma cell. *Biochemical and Biophysical Research Communications*.

[31] Akaboshi SI, Watanabe S, Hino Y (2009). HMGA1 is induced by Wnt/*β*-catenin pathway and maintains cell proliferation in gastric cancer. *American Journal of Pathology*.

[32] Pierantoni GM, Battista S, Pentimalli F (2003). A truncated HMGA1 gene induces proliferation of the 3T3-L1 pre-adipocytic cells: a model of human lipomas. *Carcinogenesis*.

[33] Caron L, Bost F, Prot M, Hofman P, Binétruy B (2005). A new role for the oncogenic high-mobility group A2 transcription factor in myogenesis of embryonic stem cells. *Oncogene*.

[34] Richter A, Hauschild G, Escobar HM, Nolte I, Bullerdiek J (2009). Application of high-mobility-group-a proteins increases the proliferative activity of chondrocytes in vitro. *Tissue Engineering—Part A*.

[35] Karlsson EK, Lindblad-Toh K (2008). Leader of the pack: gene mapping in dogs and other model organisms. *Nature Reviews Genetics*.

[36] Pittenger MF, Mackay AM, Beck SC (1999). Multilineage potential of adult human mesenchymal stem cells. *Science*.

[37] Solursh M (1989). Cartilage stem cells: regulation of differentiation. *Connective Tissue Research*.

[38] Fischer M, Skowron M, Berthold F (2005). Reliable transcript quantification by real-time reverse transcriptase-polymerase chain reaction in primary neuroblastoma using normalization to averaged expression levels of the control genes HPRT1 and SDHA. *Journal of Molecular Diagnostics*.

[39] Brinkhof B, Spee B, Rothuizen J, Penning LC (2006). Development and evaluation of canine reference genes for accurate quantification of gene expression. *Analytical Biochemistry*.

[40] Hammond SM, Sharpless NE (2008). HMGA2, MicroRNAs, and stem cell aging. *Cell*.

[41] Strem BM, Hicok KC, Zhu M (2005). Multipotential differentiation of adipose tissue-derived stem cells. *Keio Journal of Medicine*.

[42] Martinello T, Bronzini I, Maccatrozzo L (2010). Canine adipose-derived-mesenchymal stem cells do not lose stem features after a long-term cryopreservation. *Research in Veterinary Science*.

[43] Csaki C, Matis U, Mobasheri A, Ye H, Shakibaei M (2007). Chondrogenesis, osteogenesis and adipogenesis of canine mesenchymal stem cells: a biochemical, morphological and ultrastructural study. *Histochemistry and Cell Biology*.

[44] Narita M, Narita M, Krizhanovsky V (2006). A novel role for high-mobility group a proteins in cellular senescence and heterochromatin formation. *Cell*.

[45] Richter A, Lübbing M, Frank HG, Nolte I, Bullerdiek JC, von Ahsen I (2011). High-mobility group protein HMGA2-derived fragments stimulate the proliferation of chondrocytes and adipose tissue-derived stem cells. *European Cells & Materials*.

[46] Palumbo R, Sampaolesi M, De Marchis F (2004). Extracellular HMGB1, a signal of tissue damage, induces mesoangioblast migration and proliferation. *Journal of Cell Biology*.

[47] Meng E, Guo Z, Wang H (2008). High mobility group box 1 protein inhibits the proliferation of human mesenchymal stem cells and promotes their migration and differentiation along osteoblastic pathway. *Stem Cells and Development*.

[48] Zhao C, Sun G, Li S (2010). MicroRNA let-7b regulates neural stem cell proliferation and differentiation by targeting nuclear receptor TLX signaling. *Proceedings of the National Academy of Sciences of the United States of America*.

